# A multi-modal prompt-tuning method of ultrasound diagnosis for thyroid nodule

**DOI:** 10.3389/fmed.2025.1686374

**Published:** 2025-12-08

**Authors:** Xiao Xiao, Ying Zhou, Yi Zhu, Yun Li, Tingyue Qi, Wei Wang

**Affiliations:** 1 Department of Ultrasound, The Affiliated Hospital of Yangzhou University, Yangzhou, China; 2 Department of Information Engineering, Yangzhou University, Yangzhou, Jiangsu, China; 3 Department of Radiology, The Affiliated Hospital of Yangzhou University, Yangzhou, China

**Keywords:** multi-modal, prompt-tuning, ultrasound diagnosis, thyroid nodule, medical artificial intelligence

## Abstract

**Background and objective:**

Accurate diagnosis of thyroid nodules using ultrasound images heavily depends on the clinical expertise of radiologists. This reliance poses significant challenges in underdeveloped countries and regions where access to specialized medical resources is limited. Recently, Multi-modal Large Language Models (M-LLMs) have demonstrated promising potential in handling heterogeneous data, such as images and text, making them attractive candidates for automating labor-intensive diagnostic tasks. However, M-LLMs often struggle in ultrasound diagnosis of thyroid nodules for two main reasons: (1) without domain-specific fine-tuning, they are prone to generating hallucinated content, especially in classification tasks that demand expert-level decision-making; and (2) the cost and effort required for ultrasound multi-modal datasets of thyroid nodules are prohibitively high, which are essential for fine-tuning M-LLMs.

**Methods:**

We propose a novel multi-modal prompt-tuning method based on ultrasound images and textual description, which can assist radiologists in improving their diagnoses of the etiology of thyroid nodules. Our approach leverages an image encoder and a prompt-tuning framework to learn effective representations from both modalities without the need for expensive full model fine-tuning. The fused multi-modal features are then used to improve the diagnosis of thyroid nodules. These obtained features are re-input into the multi-layer perceptron (MLP) model to fuse multi-modal relationships for complementing image features and assist in the diagnosis of thyroid nodules.

**Results:**

Extensive experiments on publicly available and private enrolled datasets demonstrate that our method achieved state-of-the-art performance. Our method significantly outperformed traditional single-modality methods, with accuracy improvements of up to 40.62 over ResNet and 28.51% over AlexNet on the publicly available dataset. In contrast to other multi-modal models, our method achieved superior performance of up to 23.12% and 25.21% on accuracy and F1 score.

**Conclusions:**

Our method even surpasses all participating radiologists in accuracy, highlighting its strong potential to assist in expert-level diagnostic decision-making and provide scalable support for resource-limited clinical environments. Practically, it facilitates faster and more consistent thyroid nodule screening, thereby enhancing diagnostic efficiency.

## 1 Introduction

Thyroid Nodule is a common disease occurring in patients of all ages, with a prevalence of almost 25% in the general population (1). Referral patterns and treatment strategies for different types of thyroid nodules are all distinct; thus, accurate identification of the specific etiology is essential for subsequent medical management (2, 3). Imaging methods are the main tools for the detection, diagnosis, and follow-up monitoring of thyroid nodules, including UltraSound (US) imaging, computed tomography (CT), and magnetic resonance imaging (MRI). In contrast to other imaging modalities, the US is more convenient, economical, and radiation-free and has better resolution in characterizing nodules (4, 5). However, the diagnostic performance of ultrasound heavily depends on the expertise of radiologists, leading to challenges in the differential diagnosis of thyroid nodules, especially in many countries and regions with underdeveloped healthcare conditions, involving a huge population worldwide (6, 7).

The recent Multi-modal Large Language Models (M-LLMs) have shown remarkable capability in comprehensive understanding and processing of diverse data including images and text (8). They can achieve promising performance in visual language tasks such as image reasoning and further form user-oriented multi-modal conversation assistants, as exemplified by GPT-4 (9) and LLaVA (10). Based on the success of M-LLMs in the general domain, similar initiatives have emerged for medical applications (11). Current research is gradually transitioning from uni-modal text to incorporating multi-modal medical data, offering promises for automating the typically labor-intensive and time-consuming analytical tasks. For instance, Haider et al. proposed Dual-OptNet (12), which integrates ResNet skip connections with InceptionV3 multi-scale feature extraction and employs a dual optimization strategy using Adam and SGD, achieving high-accuracy diagnosis of multiple thyroid diseases. Moreover, SkinGPT-4 integrates multi-modal large language models with visual transformers (13), significantly enhancing diagnostic accuracy and clinical applicability in dermatology. Similarly, LLaVA-Ultra has been developed as a vision-language assistant tailored for ultrasound imaging (11), demonstrating promising results in clinical practice.

**Figure 1:**
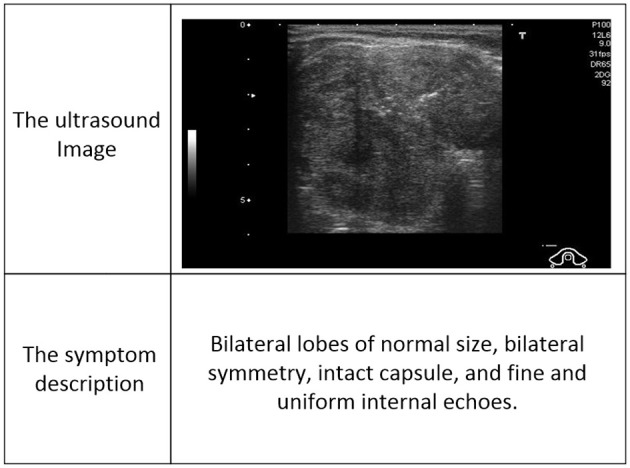
The example of image-text data from DDTI.

**Figure 2:**
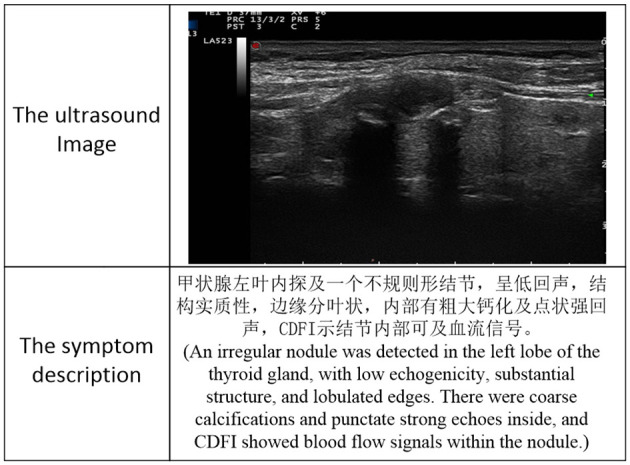
The example of image-text data from Thyroid-YZU.

Despite the effectiveness in general tasks, M-LLMs often struggle in the ultrasound diagnosis of thyroid nodules, which can be summarized as two main limitations. (1) On the one hand, without model fine-tuning, M-LLMs are prone to hallucinations, especially in specific domains where decision-making requires specialized knowledge and professional expertise, which can easily lead to a decrease in diagnosis performance. On the other hand, fine-tuned LLM is still costly in terms of computing power and time, which is expensive and labor-intensive. For example, 1.7M ultrasound images and 188k clinical texts require training times of 60 hours with four 48GB A40s (11). (2) Unlike the diverse internet data available in the general domain, large-scale professional ultrasound image-text datasets are difficult to obtain, particularly for diseases such as thyroid nodules, as illustrated in Figures 1, 2. Previous works often utilize image-text data extracted from PubMed public papers, which may be coarser and less cross-modal matched than those from primary sources. In the real world, the collection of a large-scale ultrasound multi-modal dataset of thyroid nodules is not always accessible and is costly to acquire.

To address these limitations, in this paper, we propose a novel multi-modal prompt-tuning method without the need for expensive full model fine-tuning, which can assist radiologists in improving their diagnoses of the etiology of thyroid nodules. (1) For *Limitation 1*, a SOTA pre-trained Bidirectional Encoder representation from Image Transformers (BEiT) model (14) is utilized to learn better representations of images, and the prompt-tuning model based on pre-trained BERT is leveraged to embed the textual description. Without fine-tuned M-LLMs, the obtained representations of images and texts are re-input into the multi-layer perceptron (MLP) model to fuse multi-modal relationships for complementing image features and assist in the diagnosis of thyroid nodules. (2) For *Limitation 2*, a few-shot prompt-tuning model based on the Pre-trained Language Model (PLM) is performed, and just few image-text data are collected to construct the model and optimize the verbalizer to capture the discriminative features, which can further improve the model performance. In this way, the method can learn a more accurate feature representation with only a small number of training samples and effectively improve diagnosis accuracy. Extensive experiments on both publicly available and privately enrolled datasets demonstrate that the proposed method outperforms the state-of-the-art methods, which were systematically more accurate than all the participating radiologists. In summary, the contributions of this paper are as follows:

- We propose a novel multi-modal prompt-tuning method by jointly modeling the textual and image information into the prompt embedding, which are re-input into the MLP model for improving diagnoses of thyroid nodules.
- In contrast to existing works that rely on massive professional ultrasound image-text data to fine-tune M-LLMs, the proposed method leveraged fused features to improve diagnosis accuracy without the need for expensive full model fine-tuning.
- We experimentally show that the proposed method is more robust and effective than SOTA baselines on both public and privately collected datasets.

The dataset and source code for reproducing our results are available at https://github.com/zhuyiYZU/MP-UDTN.

## 2 Related work

### 2.1 Multi-modal medical diagnosis

Early methods for multi-modal medical diagnosis primarily relied on manually designed features and traditional machine learning algorithms, which were often based on low-level image features such as texture, shape, density, and edges. For example, Gao et al. proposed a classification method for liver ultrasound images using texture analysis techniques (15), aiming to distinguish liver lesions based on texture features. Similarly, Liu et al. employed local texture analysis to extract tumor features from ultrasound images and classified them using machine learning models (16). These methods typically combined texture features such as the Gray Level Co-occurrence Matrix (GLCM) and Histogram of Oriented Gradients (HOG) with classifiers like Support Vector Machines (SVM) or Random Forests to differentiate between benign and malignant lesions. However, the performance of these methods was limited by feature selection and representation capabilities, making them insufficient for addressing complex medical scenarios.

With advancements in deep learning technologies, typical end-to-end models, e.g., Convolutional Neural Networks (CNNs), have made significant progress in multi-modal medical diagnosis (17). For example, Li et al. automatically extracted high-level semantic features from images based on CNNs, significantly improving classification accuracy for breast nodule diagnosis (18). For the classification of thyroid and breast lesions, Zhu et al. proposed a generic deep learning framework that integrated features such as lesion morphology, echotexture, edge sharpness, and surrounding tissue characteristics in ultrasound images, substantially enhancing diagnostic accuracy in distinguishing benign from malignant lesions (19). Additionally, Liu et al. introduced a method that combined transfer learning with hybrid features, integrating high-level features from deep models with traditional texture features to further improve the accuracy and generalization of thyroid nodule classification (20). Luo et al. conducted a comprehensive survey summarizing advances in pre-trained language models in the medical domain (21). The study explored methods for optimizing pre-trained models using medical-specific corpora and examined how high-level language features extracted by deep models could be integrated with domain-specific medical knowledge to enhance the accuracy and generalization of medical text processing tasks. However, these deep learning methods demonstrate deficiencies in multi-modal feature integration and cross-institutional generalization capability, consequently restricting their effectiveness when applied to complex and variable real-world clinical scenarios.

More recently, with the widespread use of pre-trained language (PLMs) models in various NLP tasks, fine-tuning models with a small amount of labeled data allows the upstream pre-trained knowledge to be fully applied to downstream tasks (22). For example, Auke et al. explored the application of soft-prompt tuning techniques for predicting lung cancer from Dutch primary care free-text medical notes (23). Zhang et al. proposed a framework combining contrastive language-image pretraining and multi-scale EEG fusion for multi-modal public health interventions, thereby improving overall effectiveness and stability (24). Furthermore, the introduction of vision pre-trained models (such as ResNet and EfficientNet) and the application of transfer learning have enabled the development of high-performance classification models, even with small-scale medical datasets. For instance, Zhou et al. proposed the SkinGPT-4 system, which innovatively integrates multi-modal large language models with visual transformers, significantly enhancing both the diagnostic accuracy and clinical utility in dermatology (13). He proposed an improved Partial Volume interpolation method for non-rigid multi-modal medical image registration, achieving higher accuracy and robustness (25). Yang et al. proposed the Adaptive Masking of Subnetworks Strategy (AMSS) to address the modality imbalance issue in multi-modal optimization (26).

Although current multi-modal methods have achieved significant improvements over traditional feature-based approaches, they generally rely on large amounts of labeled data and computationally expensive training, making them difficult to apply directly to ultrasound imaging or small-sample environments such as thyroid nodule diagnosis. In contrast, our method is specifically designed to address these practical challenges in ultrasound diagnosis. In particular, it adopts a lightweight and efficient architecture that maintains robust performance even under limited data conditions, reducing reliance on large-scale annotated datasets and computationally intensive training. Moreover, our method explicitly handles the unique characteristics of ultrasound images, including highly variable lesion morphology, low contrast, and noise interference, while enabling efficient multi-modal feature integration without requiring full fine-tuning of large pre-trained models. In contrast to existing multi-modal methods, our method provides a practical solution that is both computationally and operationally feasible for thyroid nodule diagnosis.

In summary, Table 1 provides a systematic comparison between current state-of-the-art methods and our proposed method in terms of modality type, dataset scale, and performance. We can observe that most existing methods rely on large-scale or full-set training and predominantly focus on single-modality analysis. Furthermore, our survey reveals that studies on thyroid images or ultrasound images are also largely limited to single-modality methods. In contrast, our method adopts a multi-modal prompt-tuning strategy to effectively fuse visual and linguistic features, demonstrating superior performance in few-shot scenarios.

**Table 1:** Comparison of representative methods and our method.

**Method**	**Modality**	**Dataset size**	**Perf**.
CNN-based (AlexNet, ResNet, InceptionV3)	Image	Large	Moderate
PaliGemma	Img+Text	Large	Low
BaitRadar	Img+Text	Large	High
TTCM	Ultrasound	Full set	High
USFM	Ultrasound	Full set	High
Our method	Ultrasound + Text	Few-shot	Superior

Modality refers to the type of data modality, Dataset Size refers to the relative size of the training dataset, and Perf. refers to the overall performance level.

### 2.2 Prompt-tuning

Over the past few years, prompt-tuning has effectively leveraged the potential of pre-trained models by framing downstream tasks as cloze-style problems (27). The primary components include a template and a set of label words, where the template is a background description of the current task and the label words are the high-probability vocabulary predicted by PLMs in the current context.

Recent studies have introduced hand-crafted discrete prompts, which, despite being fixed during training, demonstrated notable advantages. Such prompts allowed precise control over model behavior, enabling researchers to inject task-specific prior knowledge and linguistic intuition directly into the model (28). For example, Du et al. constructed the vocabulary of a pre-trained language model as a KD-tree and used the KNN algorithm to search for the optimal prompt template on the KD-tree (29). This method is referred to as knowledge-based average gradient search. Li et al. transformed adversarial defense into a domain adaptation problem through manually designed templates, significantly outperforming traditional fine-tuning methods and automated prompting approaches in few-shot scenarios (30).

In prompt-tuning, verbalizer refers to a mapping from labeled words to categories, which is an effective strategy to improve model performance (31). Although manually constructed verbalizers have shown promising results in certain NLP tasks, their heavy reliance on prior knowledge can lead to omissions or biases in knowledge expansion. Recently, researchers have proposed a series of methods for automatically constructing verbalizers (32). For example, Zhu et al. effectively addressed the semantic evolution challenges in short text stream classification through their innovative self-resource knowledge expansion mechanism, providing a novel pathway for NLP applications in dynamic environments (31). Specifically, they leveraged open knowledge graphs (e.g., Related Words) to retrieve vocabulary related to each topic, enhancing verbalizer performance by retaining highly relevant words and eliminating low-quality ones. Wei et al. developed a prototype-guided verbalizer construction method that extracts knowledge using pre-trained language models (KPT++) (33). This method employs prototype networks to generate embedding representations for each label in the feature space and optimizes various objective functions through contrastive learning mechanisms. Zhu et al. proposed an automatic verbalizer generation method for short-text classification (34), which considers both textual content and class name characteristics when expanding the label vocabulary space. Furthermore, in the field of medical image classification, Wu et al. introduced a novel anatomically constrained neural network designed primarily for cardiac image enhancement and segmentation tasks (35). Wang et al. proposed a direct prompt optimization method based on continuous representations, which relaxes discrete prompt optimization into a continuous space problem and improves the optimization efficiency of small models (36).

In contrast to recent prompt-tuning approaches that primarily concentrate on text-based tasks and rely on discrete templates or automatically constructed verbalizers, our method extends these concepts to a multi-modal framework specifically tailored for ultrasound diagnosis. Although prompt-tuning has been explored within the domain of medical diagnosis, existing studies have yet to fully harness the potential of multi-modal contextual information. These limitations underscore the necessity for more effective integration strategies and motivate the development of our proposed multi-modal prompt-tuning method. Distinct from existing methods that depend heavily on large-scale annotated data or overlook modality-specific challenges, our method adopts a lightweight architecture coupled with an efficient feature fusion strategy, enabling robust performance even under limited-sample conditions. Notably, our method explicitly addresses the unique challenges inherent to ultrasound imaging, thereby offering a more practical and clinically relevant solution for thyroid nodule classification.

## 3 Methodology

### 3.1 Motivation

Thyroid Nodule is a common disease occurring in patients of all ages, and accurate identification of the specific etiology is essential for subsequent medical management. The ultrasound imaging method is the main tool for the detection, diagnosis, and follow-up monitoring of thyroid nodules. However, the diagnostic performance of ultrasound images strongly relies on the clinical and professional expertise of radiologists. The unique characteristics associated with ultrasound images, such as noise, low contrast, and limited resolution, make effective utilization and classification a major challenge.

Despite the success of Multi-modal Large Language Models (M-LLMs) in medical image classification, two major challenges persist in the ultrasound diagnosis of thyroid nodules. First, M-LLMs are prone to generating hallucinated content, especially in diagnostic tasks that demand expert-level decision-making. Second, due to the complex contextual information in ultrasound images required for accurately identifying lesion regions, the cost and effort needed to build large-scale ultrasound multi-modal datasets for thyroid nodules are prohibitively high.

In this paper, a multi-modal prompt-tuning method is proposed for ultrasound diagnosis of thyroid nodules, which models both ultrasound images and symptom description texts as continuous prompt embeddings input into a pre-trained language model (PLM). Specifically, the pre-trained BEiT model is introduced to learn image features while prompt-tuning is employed to process the textual content. The resulting image and text representations are then fed into the MLP model, where the multi-modal information is fused to complement image-based features. Our method transforms the classification task into a natural language prompt, which allows the pre-trained model to learn richer features with only a small number of labeled samples. Experimental results on both publicly available and privately enrolled datasets demonstrate that the proposed method outperforms the SOTA baseline methods in terms of accuracy and robustness, offering a new approach to enhance the classification performance and application value of ultrasound images.

### 3.2 Overall architecture

The multi-modal ultrasound diagnosis for thyroid nodules can be defined as a binary classification problem, with the primary goal of determining whether the displayed ultrasound image is benign or malignant. Given the ultrasound images *P* with corresponding symptom descriptions *T*, the model will output *Y* = 0, 1 to indicate the label, where *Y* = 0 and *Y* = 1 denote that the patient is malignant and benign, respectively.

**Figure 3:**
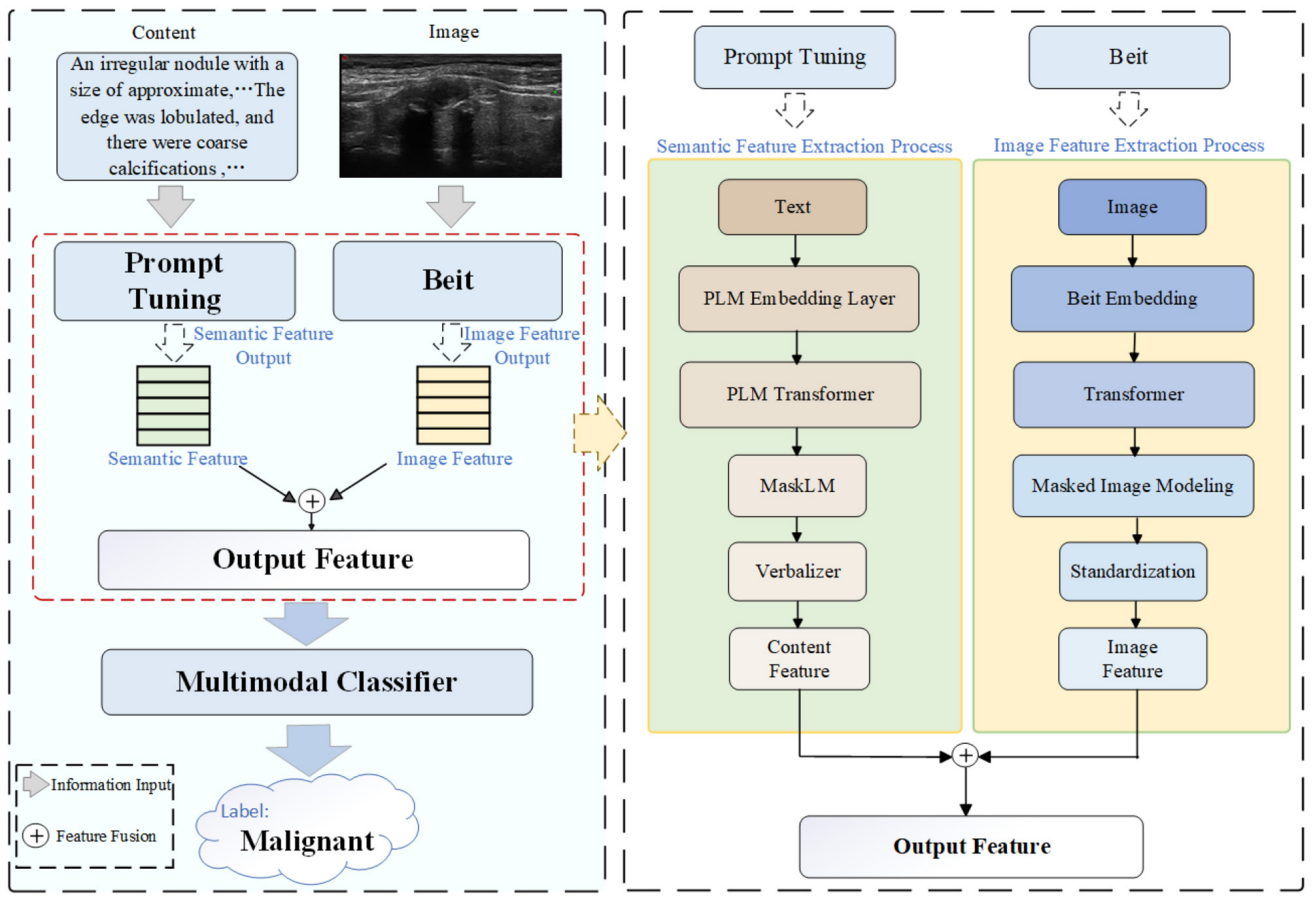
Illustration of the architecture of our method. The left part illustrates the main framework of the proposed method, while the right part provides a detailed description of the structure of the multi-modal network, which corresponds to the region indicated by the red dashed box on the left. For textual information processing, a prompt-tuning model is designed to extract semantic features from symptom description texts. For image information processing, the pre-trained model BEiT is employed to extract regional features from ultrasound images. Afterwards, the two types of features are fused and fed into a multi-modal classifier to produce the final classification result, namely benign or malignant thyroid nodules.

**Figure 4:**
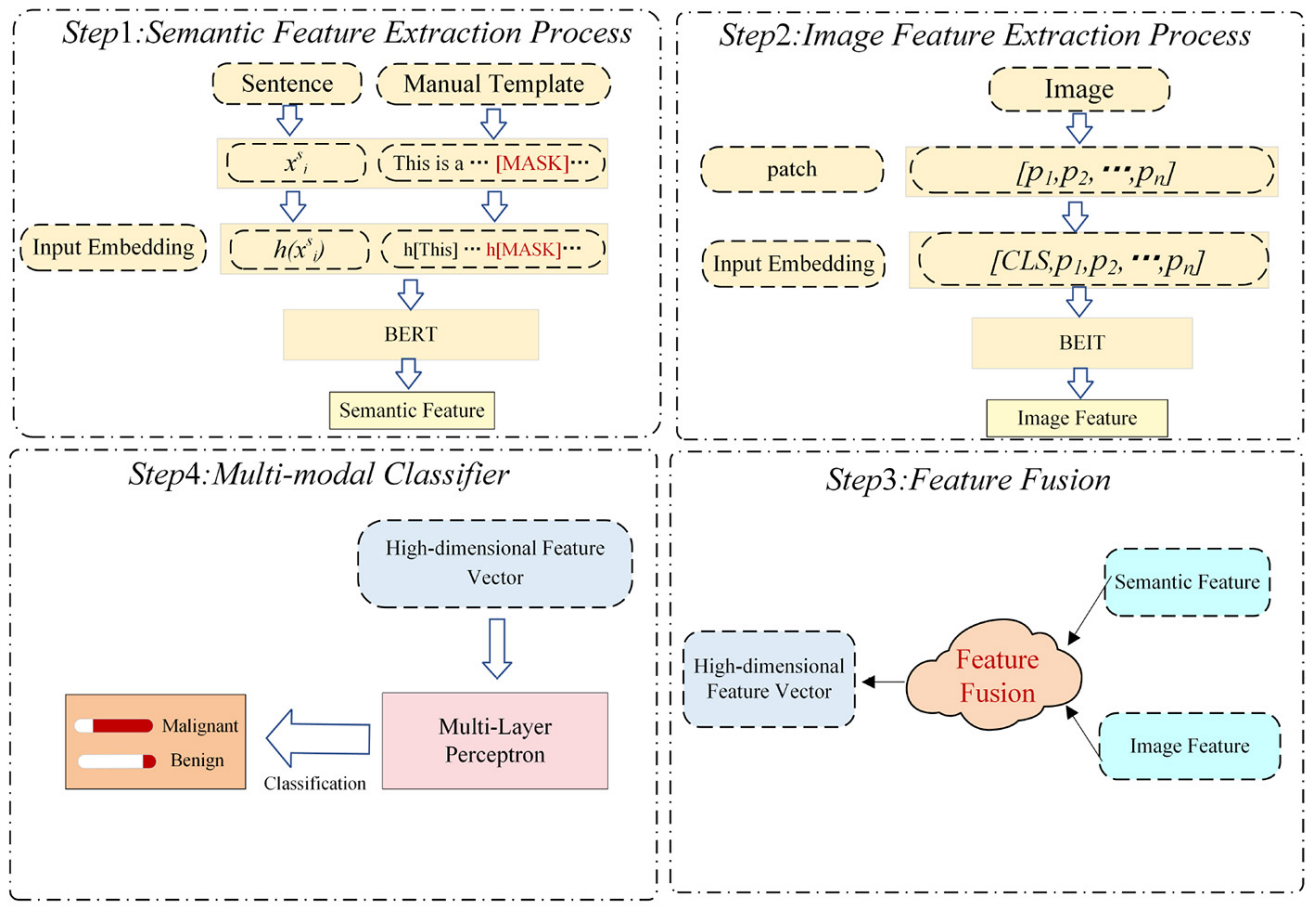
The workflow of our method, which is organized into four main steps. In the first step, the input clinical text is embedded into a predefined manual template and processed with a prompt-tuning strategy on a pre-trained BERT model to extract semantic feature representations. In the second step, the input ultrasound image is divided into patches, each embedded with positional encodings, and then processed by a pre-trained BEIT model to extract deep visual features. In the third step, semantic and visual features are aligned and integrated through a feature fusion module to form a high-dimensional multi-modal feature vector. In the fourth step, the fused features are fed into a multi-layer perceptron–based classifier to predict the probability of a nodule being benign or malignant.

We propose an innovative multi-modal prompt-tuning method, specifically designed for ultrasound diagnosis of thyroid nodules. This method jointly models textual and image information to generate a continuous prompt embedding, which is then used as input to the MLP model for classification. The overall framework is illustrated in Figure 3. The complete workflow of our method, organized into four main steps, is shown in Figure 4. The detailed algorithm description is presented in Algorithm 1. Specifically, our model consists of the following components:

- **Semantic feature extraction:** In order to understand the textual information about ultrasound description, the manually designed prompts are introduced to learn the text feature representations.
- **Image feature extraction:** For each image, we utilize the pre-trained BEiT model to extract region features. In contrast to other feature learning models like ResNet (37) and CLIP (38), BEiT can capture more abstract features and achieve better results, which has been validated in Section Experiments. Due to the information of ultrasound images being the main component in the diagnosis of thyroid nodules, the textual information can provide complementary insights to the image features to provide a better understanding of the multi-modal data. Thus, we propose a multi-modal contextual network to fuse high-order complementary information.
- **Feature fusion and prediction:** The diagnosis of thyroid nodules aims to distinguish whether each instance is benign or malignant, which is constructed on the ground of prompt-tuning to generate the predicted probability for classification.

**Algorithm 1:** A multi-modal prompt-tuning method of ultrasound diagnosis for thyroid nodule.

**Require:** Textual Symptom descriptions *T* = {*w*_1_, *w*_2_, …, *w*_*n*_}, Ultrasound Images *P* = {*p*_1_, *p*_2_, …, *p*_*m*_}, prompt tuning model *PT*.
**Ensure:** *y*∈*Y* = {benign, malignant}: the classification result.
1: **Semantic feature extraction:**
2: *X*←{*T*, [hard prompt]} // Construct hard prompt input
3: *F*_sem_←*PT*(*X*) // Extract semantic features using the prompt tuning model
4: **Image feature extraction:**
5: **for** each *p*_*i*_∈*P* **do**
6: $h_{i}^{p}\leftarrow \text{BEiT}\left(p_{i}\right)$ // Encode the image using the pre-trained BEiT model
7: **end for**
8: $F_{i}^{p}\leftarrow \text{SD}\left(h_{i}^{p}\right)$ // Standardize image features
9: $F_{\text{img}}\leftarrow \text{concatenate}\left(F_{1}^{p},F_{2}^{p},\ldots ,F_{m}^{p}\right)$ // Form the final image features
10: **Feature fusion and prediction:**
11: *F*_*c*_←concatenate(*F*_sem_, *F*_img_) // Concatenate semantic and image features
12: *y*←MLP(*F*_*c*_) // Predict using a multi-layer perceptron
13: **return** *y* // Return the classification result

### 3.3 Semantic feature extraction

To understand the textual information about ultrasound description, manually designed prompts are introduced to learn the feature representations of symptom description texts.

Given the symptom description text of thyroid nodule as *T* = {*w*_1_, *w*_2_, …, *w*_*n*_}, the label of thyroid nodules can be represented as *V*_*y*_, where these *V*_*y*_ words will be mapped to the label *Y*. For example, {malignant,abnormal,...} and {benign,normal,....} can be *V*_0_ and *V*_1_ as malignant and benign tumor categories, respectively. Thus, the probability of filling each word *v* in *V*_*y*_ into the [*mask*] token can be expressed as *p*([*mask*] = *v*∈*V*_*y*_∣*T*), the classification task is transformed into calculating the probability of the label words as Equation 1:


$$\begin{array}{l} p\left(y|x\right)=p\left(\left[mask\right]=v\epsilon V_{y}|T\right) \end{array}$$ (1)

Notably, prompt-tuning can be categorized into two main types: hard prompts and soft prompts. In our experiments, we designed various hard and soft prompt templates to process the text. Through comparison, we found that hard prompts yielded better results, which has been demonstrated in Section Experiments. The input is constructed by concatenating the symptom description text *T* with the hard prompt:


$$\begin{array}{l} X=\left\{T,\left[\text{Hard Prompt}\right]\right\} \end{array}$$ (2)

Then, the semantic features are extracted using the prompt tuning model:


$$\begin{array}{l} F_{\text{sem}}\leftarrow \text{PT}\left(X\right) \end{array}$$ (3)

This semantic representation serves as a critical component in the subsequent multi-modal fusion process and contributes directly to the final classification task.

### 3.4 Image feature extraction

In recent years, vision transformers have made significant progress, with models such as ViT, Swin Transformer, and BEiT demonstrating strong capabilities in visual representation learning. ViT directly applies transformer blocks to image patches. However, it generally requires large-scale datasets for effective training. Swin Transformer introduces hierarchical feature maps and shifted window mechanisms. This design improves scalability but also increases architectural complexity. In contrast, BEiT leverages masked image modeling with discrete tokens, enabling more efficient capture of high-level semantic representations from limited data. Considering the relatively small size of ultrasound datasets and the demand for robust semantic feature extraction, BEiT achieves a favorable balance between performance and data efficiency. Therefore, it is adopted as the feature extraction module in this study. Based on this, we employ a pre-trained BEiT model to extract features from each ultrasound image. In contrast to other feature learning models, BEiT demonstrates a significant advantage in capturing more abstract and semantic image representations, a superiority that is validated in our experimental section.

Given P = {*p*_1_, *p*_2_..., *p*_*i*_}, for each image *P*_*i*_, we first use the BEiT encoder for feature extraction as follows:


$$\begin{array}{l} P_{i}=Beit\text{\_}Encoder\left(p_{i}\right) \end{array}$$ (4)

Next, the features of each image are projected into a lower-dimensional space, making the features more compact and facilitating subsequent processing. The projected features can be represented as:


$$\begin{array}{l} P_{i}^{\prime }=f_{p}\left(P_{i_{i}}\right) \end{array}$$ (5)

Then, to mitigate the impact of outliers and noise, normalization is performed on the image features:


$$\begin{array}{l} P_{i}^{N}=\frac{P_{i}^{\prime }-\mu }{\sigma } \end{array}$$ (6)

Finally, after obtaining the feature representations of all images, the image features are concatenated based on the processed representations of each image. The features of all images $F_{1}^{p}$, $F_{2}^{p}$, …,$F_{m}^{p}$ are merged into a unified feature set *F*_img_ as Equation 7:


$$\begin{array}{l} F_{\text{img}}=\text{concatenate}\left(F_{1}^{N},F_{2}^{N},\ldots ,F_{m}^{N}\right) \end{array}$$ (7)

### 3.5 Feature fusion and prediction

After extracting the textual and image features, we fuse the multi-modal information to enhance the model's ability to distinguish between benign and malignant thyroid images.

#### 3.5.1 Feature fusion

To fully exploit the complementarity between textual and image information, we concatenate the features from the two modalities. Let the extracted semantic feature be denoted as *F*_sem_, and the normalized image feature be denoted as *F*_img_. The final fused representation is formulated as Equation 8:


$$\begin{array}{l} F_{c}=\text{Concatenate}\left(F_{\text{sem}},F_{\text{img}}\right) \end{array}$$ (8)

In the feature fusion stage, we concatenate the 768-dimensional text feature vector *F*_sem_ obtained from BERT with the 512-dimensional image feature vector extracted by BEiT. The resulting high-dimensional vector *F*_img_, which contains richer information, is then used for subsequent prediction.

#### 3.5.2 Prediction

We embed the high-dimensional fused feature vector *F*_*c*_ obtained above into a predefined manual template and feed it into the pre-trained language model (PLM). The specific design of the template will be discussed in Section 4.6. Then, based on a defined set of label words, the prediction probability at the [MASK] position is projected to the task labels. During training, the cross-entropy loss at the [MASK] position is minimized. During inference, the label word with the highest probability at the [MASK] position is selected as the final classification result.

The fused feature *F*_*c*_ is then fed into a multi-layer perceptron (MLP) for classification. The MLP consists of multiple fully connected layers, combined with nonlinear activation functions, to learn complex cross-modal feature interactions. The classification output is computed as follows:


$$\begin{array}{l} Y=\text{MLP}\left(F_{c}\right) \end{array}$$ (9)

where *Y*∈{0, 1} represents the predicted label, with 0 indicating a malignant nodule and 1 indicating a benign nodule.

## 4 Experiments

### 4.1 Datasets

In the experiments, we used two ultrasound datasets of thyroid nodules, with statistical information provided in Table 2. The details are as follows:

**Table 2:** Statistics of the dataset.

**Statistic**	**DDTI**	**Thyroid-YZU**
# total samples	390	279
# train samples	230	165
# test samples	160	114
#.avg images	390	279

“#” and “#.avg images” denote “the number of” and “the number of images per dataset.”

**DDTI** ^1^**:** It consists of 390 thyroid ultrasound images, categorized into benign and malignant conditions. Among these, 144 images are of benign thyroids, and 246 images are of malignant thyroids. The dataset provides numerical information for each image, such as thyroid volume and nodule size, but lacks explicit descriptions of symptoms.

**Thyroid-YZU** ^2^**:** This dataset contains 279 ultrasound images of thyroid tumors, aimed at distinguishing between benign and malignant tumors. Of these, 181 images are of benign tumors, and 98 images are of malignant tumors. The images represent patients across different age groups and genders, ensuring diversity and representativeness of the data. This dataset also provides numerical information for each image, similar to the DDTI dataset.

Notably, given the relatively small size of the two datasets used in this study, we explored the possibility of incorporating external large-scale datasets, such as TCIA (39) and TCGA-Thyroid (40). However, both datasets predominantly comprise computed tomography (CT) or other imaging modalities of thyroid cancer, and do not include ultrasound images. As our research specifically targets ultrasound-based diagnosis of thyroid nodules, these datasets are unsuitable for the current experimental setting and therefore cannot be utilized for model training or validation.

### 4.2 Baselines

To validate the effectiveness of our method, we conducted experiments with several advanced image and multi-modal methods that have achieved significant results in auxiliary diagnosis tasks:

**AlexNet** (41): An improved convolutional neural network that utilizes multiple convolutional and pooling layers, enabling the model to learn more complex feature representations. It has demonstrated excellent performance in medical image classification tasks such as lesion detection and tissue classification.

**ResNet** (42): A deep convolutional neural network that incorporates residual modules to address the gradient vanishing problem in deep networks, allowing for more stable learning of deep feature representations. It has shown remarkable performance in lesion detection and few-shot medical image classification tasks.

**InceptionV3** (43): A model designed for various pathological feature classification tasks, which extracts multi-scale features by combining convolution kernels of different sizes at the same layer. This approach is better suited to handle the complexity of medical images.

**Paligemma** (44): A multi-modal vision-language model developed and released by Google, offering a wide range of capabilities to combine textual and visual information. Through fine-tuning, it achieves efficient performance in specific tasks.

**BaitRadar** (45): A multi-modal method that combines deep learning techniques to identify target features by analyzing multiple attributes. It utilizes a combination of various reasoning models, enabling the processing and understanding of the interaction between visual and textual information, offering an effective solution for cross-modal learning in medical image analysis.

**Ours-ResNet** (37): In our method, we adopted ResNet-50 as the image features learning module. With its residual structure and deep feature extraction capabilities, ResNet demonstrates good generalization performance in medical image classification, accurately capturing pathological features in medical images.

**Ours-CLIP** (46): In our method, we replace the image features learning module with the CLIP model. CLIP leverages contrastive learning between visual and textual data to achieve cross-modal understanding, which enhances classification performance in multi-modal scenarios, especially in tasks where textual information aids in identifying lesion features.

### 4.3 Experiment settings

#### 4.3.1 Implementation details

In the experiments, we divided both original datasets into 60% for training and 40% for testing. During the construction of training samples, we randomly selected *K* training instances (*K* = 5, 10, 20) and constructed a validation set of equal size for each group of training samples. To mitigate the influence of limited training samples on the classification task and considering the number of datasets, we designed training sets for neural network-based models. Specifically, the training sample sizes for deep methods were set to 50, 100, and 200.

For the baseline methods, we adhered to the initial configurations of each model in the experiments. For the prompt-tuning-based fine-tuned models, the learning rate was set to 2e-5, and the batch size was set to 16 during training. To further optimize the model's performance, we performed a validation step during training and fine-tuned the model's hyperparameters according to specific criteria. All few-shot experiments were conducted over 10 training epochs to ensure sufficient training time and obtain stable results. Additionally, we deployed the open-source large model Paligemma locally for medical image classification using a prompt-based dialogue approach. It is important to note that we transformed the classification task into a question-and-answer format solely by constructing prompts without fine-tuning the model.

All experiments were conducted on the following hardware configuration: an NVIDIA GeForce RTX 3090 Founders Edition GPU, an Intel(R) Core(TM) i9-10980XE CPU (with a clock speed of 3.00 GHz), and 125 GB of memory. The experiment environment used Python version 3.9.16 and PyTorch version 1.7.

#### 4.3.2 Evaluation metrics

To comprehensively evaluate the effectiveness of our method, four primary evaluation metrics are adopted in the experiments, including Accuracy, Precision, Recall, and F1-score. These metrics offer a comprehensive assessment of model performance from various perspectives, with their specific definitions as follows:

**Accuracy (Acc)**: The proportion of correctly predicted samples to the total number of samples, serving as a key indicator of the model's overall classification ability.

**Precision (Pre)**: The proportion of actual positive samples among those predicted as positive, reflecting the model's accuracy in predicting positive samples.

**Recall (Rec)**: The proportion of correctly predicted positive samples among all actual positive samples, indicating the model's ability to capture positive samples.

**F1-score (F1)**: The harmonic mean of precision and recall, providing a balanced evaluation of the model's accuracy and recall capability. It is commonly used as an evaluation metric for multi-class classification tasks.

### 4.4 Main results

The detailed experimental results of all datasets are shown in Table 3. Notably, to ensure the accuracy of the classification results, we obtained the final experimental results three times and took the average value. From these experimental results, we have made the following observations:

**Table 3:** The result on two datasets.

**Datasets**	**Methods**	**5-shot**				**10-shot**				**20-shot**			
		**Acc**	**Pre**	**Rec**	**F1**	**Acc**	**Pre**	**Rec**	**F1**	**Acc**	**Pre**	**Rec**	**F1**
DDTI	AlexNet	54.34	45.48	47.00	46.20	55.86	56.78	54.00	55.32	62.95	74.72	58.50	64.92
	ResNet	42.50	45.84	28.80	35.60	53.50	44.78	79.40	57.40	58.00	52.15	70.15	59.74
	InceptionV3	79.69	**89.34**	59.38	59.83	82.81	84.09	67.71	70.85	81.25	90.00	62.50	64.44
	PaliGemma	25.89	56.72	53.72	25.29	25.89	56.72	53.72	25.29	25.89	56.72	53.72	25.29
	BaitRadar	60.00	57.00	60.00	56.00	65.00	70.00	65.00	63.00	77.25	73.00	72.00	72.00
	Ours-Resnet	60.00	60.25	60.67	59.69	60.62	61.29	61.71	60.43	85.62	**89.43**	82.29	83.85
	Ours-CLIP	81.87	84.00	78.64	79.82	82.50	84.47	79.42	80.60	84.37	88.69	80.72	82.28
	Ours	**83.12**	85.55	**79.94**	**81.21**	**84.37**	**88.69**	**80.72**	**82.28**	**86.25**	89.05	**83.33**	**84.76**
Thyroid-YZU	AlexNet	61.00	51.73	61.00	51.66	57.97	73.60	63.00	54.10	64.49	71.22	67.05	61.40
	ResNet	52.91	59.00	45.00	50.00	63.45	61.74	61.09	60.52	73.05	**78.84**	74.06	75.23
	InceptionV3	61.72	67.42	52.35	60.16	66.71	49.00	50.30	34.33	58.59	66.08	46.48	46.59
	PaliGemma	29.03	24.36	23.31	23.81	29.03	24.36	23.31	23.81	29.03	24.36	23.31	23.81
	BaitRadar	63.63	40.00	64.00	49.00	65.00	68.00	65.00	62.00	72.22	72.00	72.00	71.00
	Ours-Resnet	62.28	65.17	63.77	61.78	61.40	63.95	62.81	60.97	67.54	77.45	70.00	65.90
	Ours-CLIP	67.54	74.67	69.69	66.40	63.15	64.93	64.26	62.97	65.78	65.49	65.44	65.46
	Ours	**69.29**	**76.89**	**71.46**	**68.21**	**70.17**	**77.36**	**72.27**	**69.22**	**77.19**	77.27	**77.48**	**77.16**

The bolder ones mean better.

Firstly, when the number of training samples in the dataset increased from 5 to 20, the classification performance of all the methods improved. The results demonstrate the effectiveness of increasing training data in improving the accuracy of ultrasound diagnosis for thyroid nodules.

Secondly, we can observe that neural network-based methods, such as AlexNet and ResNet (using only image data), perform effectively with a large number of training samples. However, they were outperformed by the deep learning model InceptionV3, which is specifically designed for medical image detection. This model exhibited excellent performance when the training sample size was large.

Furthermore, the results indicate that the performance of Paligemma is relatively poor. While Paligemma excels in handling objective questions with clear, standardized answers, it faces challenges with professional questions without fine-tuning, which is often influenced by specific features. Meanwhile, BaitRadar, a multi-modal method introduced for medical image analysis, experiences performance degradation when applied to ultrasound diagnosis for thyroid nodules. This degradation can be attributed to the disparity in content structure and attributes, especially in the few-shot learning scenarios without fine-tuning M-LLMs.

In addition, our methods and the corresponding variants (Ours-ResNet and Ours-CLIP) all achieved promising and stable performance in most cases. The results highlight the effectiveness of our designed multi-modal framework for the diagnosis of thyroid nodules in scenarios with limited data. Notably, when the model of image features learning transforms from the deep neural network (e.g., ResNet) to a pre-trained model (e.g., CLIP and BEiT), our method exhibited improved performance in classification, which suggests that large-scale pre-trained multi-modal models can learn rich information from images.

Finally, our proposed method, which integrates text and image features, achieved better results than all the baselines across most cases and datasets. We explored various multi-modal models for processing image features and found that the BEiT model achieved the best results, surpassing ResNet and CLIP models. Therefore, we selected the BEiT model as the module for handling image features.

In terms of these experimental results, we performed the Friedman Test for accuracy on these two datasets, respectively. In the statistical test, we use different methods as the grouping variable. The value of asymptotic significance is 0.014, 0.013, 0.012, and 0.010, 0.017, 0.011 for 5-shot, 10-shot, and 20-shot in DDTI and Thyroid-YZU datasets, respectively, which are all less than α = 0.05. Hence, the results confirm that the observed improvements are statistically significant (*p* < 0.05) across both datasets.

In addition to the quantitative improvements, we further analyzed whether the observed gains are clinically meaningful. Our method consistently achieved higher accuracy and F1 scores than both baseline models. From a clinical perspective, even modest improvements in diagnostic accuracy can have significant impact, as each percentage gain translates into fewer missed malignant nodules or unnecessary interventions for benign cases. The reduction of false negatives is particularly critical, as early and accurate detection of malignant nodules directly influences treatment planning and patient prognosis. Similarly, lowering false positives reduces unnecessary biopsies and patient anxiety, thereby improving overall care quality. These findings suggest that the superiority of our method is not only statistically significant but also provides tangible clinical benefits, supporting its potential adoption as a practical decision-support tool in routine thyroid nodule assessment. The details are presented as quoted below.

### 4.5 Additional baselines: full-data models

To provide a more comprehensive evaluation, we conducted additional comparisons with two state-of-the-art models that were trained on the full dataset. It is important to emphasize the fundamental difference in the learning settings. The baseline models were trained on the entire training set, while our method operates in a few-shot learning scenario using only K (5, 10, 20) samples per class. This comparison aims not for direct performance parity but to highlight the data efficiency of our approach in scenarios where labeled data is scarce. The two full-data baseline models are described as follows:

- **Thyroid Tumor Classification Model (TTCM)** ^3^: A vision transformer (ViT)-based model fine-tuned on thyroid ultrasound images for binary classification of malignant and benign tumors. By leveraging self-attention to capture global image features, it demonstrates superior performance over conventional CNNs and serves as a representative baseline for thyroid tumor classification tasks.
- **UltraSound Foundation Model (USFM)**: USFM is a universal ultrasound foundation model designed for label-efficient image analysis across different tasks and organs (47). It is pre-trained on a large-scale dataset of ultrasound images. For a fair comparison in our thyroid nodule classification task, we fine-tuned the USFM model using the full training sets.

The comparative results are summarized in Table 4. Despite having access to only a minuscule fraction of the training data (20 shots vs. full training set), our proposed method significantly outperforms both TTCM and USFM on both datasets. For instance, on the DDTI dataset, our method achieves an accuracy of 86.25%, compared to 66.84% for TTCM and 60.20% for USFM. A similar substantial performance gap is observed on the Thyroid-YZU dataset.

**Table 4:** Performance comparison of different methods on two datasets.

**Datasets**	**Methods**	**Acc**	**Pre**	**Rec**	**F1**
DDTI	TTCM	66.84	64.09	59.14	58.54
	USFM	60.20	60.34	60.15	55.52
	Ours	86.25	89.05	83.33	84.76
Thyroid-YZU	TTCM	60.97	68.62	67.45	60.89
	USFM	62.90	62.33	62.85	59.04
	Ours	77.19	77.27	77.48	77.16

Bold values indicate the best results.

The observed performance gap underscores a key contribution of our work. While models like TTCM and USFM can achieve high performance when sufficient labeled data is available for fine-tuning, their performance degrades significantly when such data is scarce, as simulated in our few-shot setting. In contrast, our multi-modal prompt-tuning method effectively leverages pre-trained knowledge from both visual (BEiT) and linguistic (PLM) domains through a carefully designed fusion strategy. This allows it to achieve robust and high-performing diagnoses with minimal requirement for labeled training data. These results demonstrate the strong potential of our method for practical medical applications, where collecting large, expert-annotated datasets is often costly and time-consuming.

### 4.6 Ablation study

To investigate the influence of different modules in our proposed method on ultrasound diagnosis for thyroid nodules, we conducted ablation experiments, with the results shown in Table 5. Specifically, “-image” indicates that we removed the image feature module and trained the model using only the enhanced prompt learning method. “-text” means we omitted the text of symptom description, training the model using only the ultrasound images.

**Table 5:** The influence of different modules in our method on the results.

**Datasets**	**Modules**	**Acc**	**Pre**	**Rec**	**F1**
DDTI	-text	84.37	88.69	80.72	82.28
	-image	64.37	61.71	53.12	57.07
	ours	**86.25**	**89.05**	**83.33**	**84.76**
Thyroid-YZU	-text	73.24	75.48	74.55	73.38
	-image	61.81	63.79	61.73	62.04
	ours	**77.19**	**77.27**	**77.48**	**77.16**

Bold values indicate the best results.

By comparing these two ablation experiments, “Ours” achieved the best performance, indicating that the accompanying text in ultrasound diagnosis can serve as auxiliary information to enhance classification performance. Relying solely on image or text information cannot yield optimal results, which aligns with the core idea of our method. Furthermore, “-text” achieved better performance than “-image,” which validated that image feature representations play a more important role in ultrasound diagnosis for thyroid nodules.

In terms of these experimental results, we conduct the Friedman Test as a significance test over both datasets for Acc, Pre, Rec, and F1, respectively. In the statistical test, we use different methods as the grouping variable. Our method has significant superiority as compared with all variants on both datasets. According to the experimental results, we can get that at significance level α = 0.05, the value of asymptotic significance of Acc is 0.017 and 0.010, the value of Pre is 0.014 and 0.014, the value of Rec is 0.022 and 0.014, and the value of F1 is 0.010 and 0.010, respectively, which are all less than α = 0.05. Hence, the results of the significance test demonstrate that there is a significant difference among different methods on all datasets.

### 4.7 Influence of the templates

To investigate the influence of different templates in prompt-tuning on classification results, we designed five manually crafted prompt templates, which have been applied in two different datasets, as detailed in Tables 6, 7, respectively. Some recent works consider that soft templates can be automatically generated to avoid time-consuming and labor-intensive processes, we introduce the soft prompt method as compared to templates. The experimental results for each template are presented in Table 8.

**Table 6:** The different hand-crafted templates in the experiments.

**ID**	**Template**
1	This is a description where the diagnosis of
	{“MASK”} is conveyed: {“placeholder”: “text_a”}
2	A {“MASK”} which is contains:
	{“placeholder”: “text_a”}
3	The {“MASK”} is linked with
	{“placeholder”: “text_a”}
4	The {“MASK”} is associated to the description of
	{“placeholder”: “text_a”}
5	The {“placeholder”: “text_a”}
	is related to {“MASK”}

**Table 7:** The different hand-crafted templates in the experiments.

**ID**	**Template**
1	这是一个关于肿瘤的描述, 其中传达了
	{~mask~} 的诊断信息: {~placeholder~: ~text_a~}
2	一种 {~mask~} 超声
	{~placeholder~: ~text_a~}。
3	这个 {~placeholder~: ~text_a~} 的
	主题是关于 {~mask~} 的
4	{~placeholder~: ~text_a~}
	是关于 {~mask~} 的
5	有关 {~placeholder~: ~text_a~} 的
	主题是 {~mask~}

**Table 8:** The Accuracy and F1-scores results of all the templates on two datasets.

**Datasets**	**TemplateID**	**Acc**	**Fl**
DDTI	temp0	86.25	84.76
	temp1	78.75	78.47
	temp2	73.75	73.24
	temp3	76.87	76.61
	temp4	75.00	74.21
	soft	83.75	81.66
	Avg	77.62	76.83
Thyroid-YZU	temp0	77.19	77.16
	temp1	66.66	64.52
	temp2	68.42	67.61
	temp3	66.66	64.52
	temp4	68.42	66.68
	soft	71.92	71.92
	Avg	69.87	68.73

The bolder ones mean better.

From the experimental results, it is evident that templates play a crucial role in helping the model better understand textual content, thereby effectively improving classification performance. It is worth noting that soft prompts fail to outperform manually crafted prompts. Experimental results indicate that while hand-crafted prompts designed for a specific dataset may not be suitable for all datasets, they consistently outperform the average performance of soft templates. Despite soft prompts can be generated without labor, manual templates can be more competitive and superior compared to soft templates due to the incorporation of rich domain expertise.

In addition, we attribute the superior performance of manual prompts partly to the integration of domain-specific medical knowledge. For example, radiology terminology and structured clinical expressions embedded in handcrafted prompts align closely with how clinicians describe and interpret ultrasound findings. This alignment provides clearer semantic guidance to the model, reduces ambiguity, and enhances its ability to focus on clinically meaningful features, thereby contributing to the improved performance of manual prompts over soft prompts. Therefore, we opted for manual templates to construct the input.

### 4.8 Parameter sensitivity

**Figure 5:**
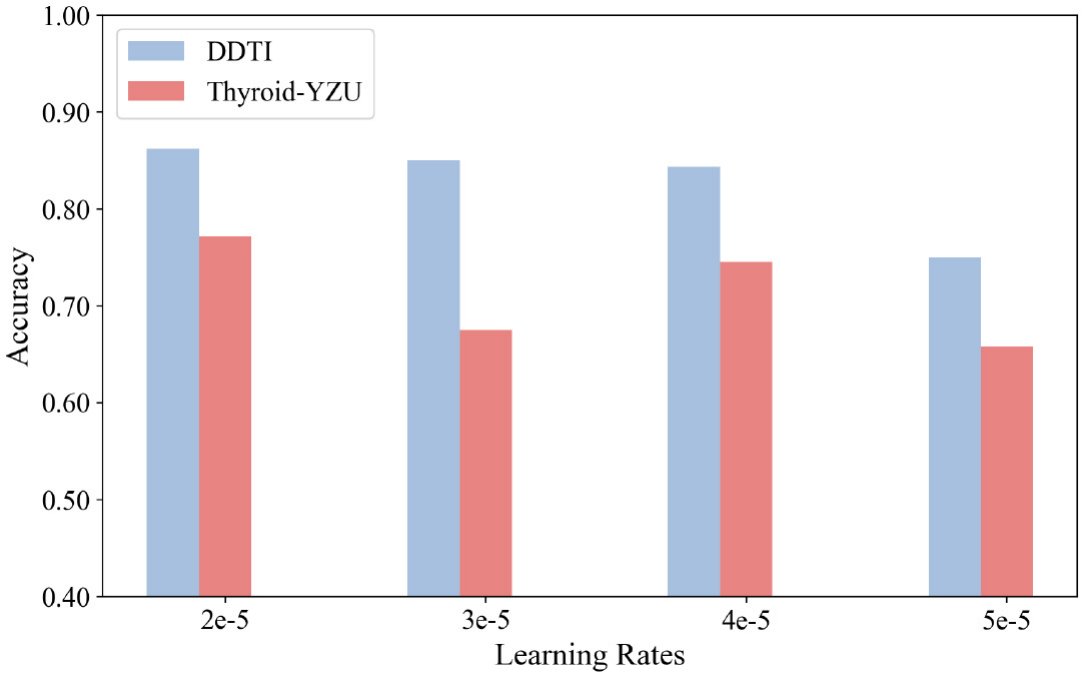
Effects of different learning rates over three datasets.

**Figure 6:**
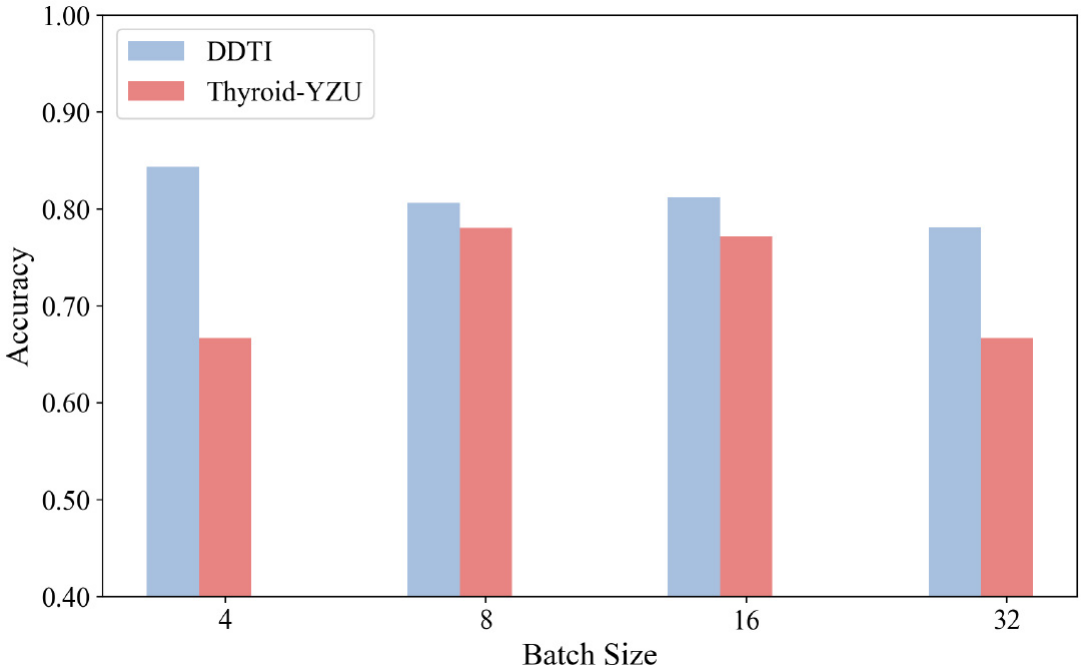
Effects of different batch sizes over three datasets.

The influence of various parameters, including batch size and learning rate, was evaluated through experiments, as shown in Figures 5, 6. From the experimental results, the learning rate was set to 2e-5, and the batch size was set to 16. The results further indicate that different datasets exhibit varying sensitivity to learning rates. The model's performance fluctuated under different learning rates, which could be attributed to adjustments in the learning rate, as it influences the convergence speed of training and thereby affects model performance. Additionally, the results demonstrate that increasing the batch size within an appropriate range can improve the model's performance across all datasets. This improvement is likely because larger batch sizes reduce gradient noise during training and effectively leverage the parallel processing capabilities of GPUs, enabling the model to converge more efficiently.

Based on these experimental findings, we determined the optimal parameter settings for our study. We chose a learning rate of 2e-5 and a batch size of 16 as the default settings for our experiments. These values represent a balance between stability and efficiency: the learning rate of 2e-5 ensures steady convergence while avoiding oscillations or divergence observed at higher rates, whereas the batch size of 16 provides sufficient gradient stability without introducing excessive computational cost. These settings have also been widely adopted in related studies, further supporting their suitability. In our context, they consistently delivered robust and reproducible performance across both datasets, confirming their appropriateness for ultrasound-based thyroid nodule diagnosis.

### 4.9 The influence of different PLMs

To evaluate the influence of different pre-trained language models (PLMs) on the experimental results, we conducted additional experiments by replacing the bert-base-cased in the DDTI method with other PLMs, including RoBERTa and stsb-roberta-large. Similarly, in the Thyroid-YZU method, we replaced the bert_base_chinese with the RoBERTa and stsb-roberta-large. The experimental results are detailed in Table 9.

**Table 9:** The Accuracy and F1-scores results by replacing different PLMs.

**Datasets**	**Model**	**Acc**	**Fl**
DDTI	bert-base-cased	**86.25**	**84.76**
	roberta	84.37	82.28
	stsb-roberta-large	85.00	82.90
Thyroid-YZU	bert_base_chinese	**77.19**	**77.16**
	roberta	73.68	73.48
	stsb-roberta-large	71.92	71.03

Bold values indicate the best results.

From the results, it can be observed that these methods exhibit strong competitiveness compared to other baseline methods. This demonstrates that our prompt-tuning-based approach effectively works across different PLMs and further validates its ability to mitigate the biases and limitations of PLMs. Notably, among all PLMs, bert-base-cased achieved the best performance on the DDTI dataset, while bert_base_chinese achieved the best performance on the Thyroid-YZU dataset.

Based on these experimental findings, we further analyzed the rationale for choosing BERT. The selection of BERT is due not only to its strong performance in experiments but also to its proven effectiveness in capturing contextual dependencies and subtle semantic nuances in clinical text descriptions. In contrast to traditional word embedding methods or lightweight pre-trained language models, BERT employs a bidirectional transformer architecture to construct rich language representations, which is particularly valuable for ultrasound diagnostic reports that often contain short but information-dense phrases. This capability enables more accurate extraction of discriminative textual features, thereby providing stronger complementary information for multi-modal fusion in thyroid nodule diagnosis. Therefore, choosing BERT is both empirically and theoretically justified for the multi-modal prompt-tuning method.

### 4.10 The influence of different fusion strategies

To thoroughly examine the role of multi-modal integration and the robustness of our feature fusion strategy, we compared our standard concatenation strategy (denoted as Concat) against three alternative strategies: Dimensionality Reduction (DimRed), Feature Interpolation (Interp), and Position Swapping (Swap). All experiments were conducted under the 20-shot setting on both datasets.

- Concat (Ours): It directly concatenates textual and image features, preserving complete modality information and enabling richer cross-modal interactions.
- DimRed: It projects text and image features into a shared 512-dimensional latent space, applies a gating mechanism to adaptively balance contributions, and then projects back to the original dimension. This strategy reduces redundancy but causes partial semantic loss.
- Interp: It introduces a residual difference-based fusion. After projecting text and image features into the same latent space, Interp was computed and combined with the original features through a gating mechanism. This strategy emphasizes the discrepancies between modalities but sometimes amplifies noise, leading to reduced stability.
- Swap: reverses the concatenation order of text and image features, allowing us to test whether feature positioning affects performance.

As shown in Table 10, the proposed Concat strategy consistently achieves the best results on both datasets. On DDTI, it obtains the highest accuracy (86.25%) and F1 score (84.76%), while on the Thyroid-YZU dataset, it also surpasses alternative strategies with an accuracy of 77.19% and an F1 score of 77.16%. These results highlight that directly concatenating textual and visual features allows the model to fully preserve modality-specific information and capture richer cross-modal interactions.

**Table 10:** Performance comparison of different fusion strategies on two datasets.

**Dataset**	**Strategy**	**Acc**	**Prec**	**Rec**	**F1**
DDTI	DimRed	76.41	74.69	74.97	74.82
	Swap	78.72	86.00	71.47	72.88
	Interp	76.41	81.58	69.06	70.03
	Ours	86.25	89.05	83.33	84.76
Thyroid-YZU	DimRed	67.41	66.24	67.39	66.25
	Swap	72.22	70.89	72.27	71.11
	Interp	67.78	65.56	59.34	58.62
	Ours	77.19	77.27	77.48	77.16

DimRed, Dimensionality Reduction; Swap, Position Swapping; Interp, Feature Interpolation. Bold values indicate the best results.

In comparison, DimRed suffers from performance degradation due to information loss during dimensionality reduction, while Interp emphasizes cross-modal discrepancies but amplifies noise, leading to lower stability, especially on Thyroid-YZU (F1 of 58.62%). Swap yields results close to Concat but remains slightly inferior, suggesting that the concatenation order has some impact but is not the determining factor. Overall, the experiments confirm that maintaining complete modality information through direct concatenation provides the most robust and reliable fusion strategy for our task.

## 5 Conclusions and future work

In this paper, we propose a novel multi-modal prompt-tuning method to assist in the diagnosis of thyroid nodules. Existing multi-modal large language models (M-LLMs) are prone to hallucinations without domain-specific fine-tuning, while the acquisition of large-scale ultrasound multi-modal datasets for thyroid nodules remains costly and labor-intensive, posing significant challenges for fine-tuning. To address these issues, we jointly model textual and image information into a continuous prompt embedding as the input of pre-trained language models (PLMs), leveraging a state-of-the-art BEiT model to extract rich visual features and a prompt-tuning model to embed textual descriptions. Experimental results demonstrate that our approach consistently outperforms state-of-the-art baselines across all datasets.

Although this study achieved promising results in ultrasound-based diagnosis of thyroid nodules, there remains room for further improvement. First, given the relatively limited dataset size, potential exists to improve model robustness and generalization ability to unseen data. Second, lack of multi-center external validation may, to some extent, limit model applicability across diverse clinical settings, devices, and acquisition protocols. To address these issues, future work will focus on exploring domain adaptation and data augmentation to enhance robustness and generalizability. In addition, clinical translation requires attention to model interpretability and system integration, where interpretability could be further strengthened to increase clinical trust, and integration should ensure user-friendly interfaces and regulatory compliance. Therefore, future efforts will concentrate on explainable artificial intelligence, interface optimization, and large-scale validation to facilitate broader clinical application of the proposed method.

## Data Availability

The original contributions presented in the study are included in the article/supplementary material, further inquiries can be directed to the corresponding author.

## Footnotes

1
https://www.kaggle.com/datasets/dasmehdixtr/ddti-thyroid-ultrasound-images

2
**https://github.com/zhuyiYZU/MP-UDTN**

3
https://huggingface.co/SerdarHelli/ThyroidTumorClassificationModel
